# Islet transplantation from a nationally funded UK centre reaches socially deprived groups and improves metabolic outcomes

**DOI:** 10.1007/s00125-015-3554-3

**Published:** 2015-03-26

**Authors:** Shareen Forbes, Neil W. A. McGowan, Kirsty Duncan, Debbie Anderson, Janet Barclay, Donna Mitchell, Kevin Docherty, David Turner, John D. M. Campbell, John J. Casey

**Affiliations:** 1 Islet Transplant Unit, Royal Infirmary of Edinburgh, Edinburgh, UK; 2 Endocrinology Unit, University/BHF Centre for Cardiovascular Science, University of Edinburgh, Queen’s Medical Research Institute, 47 Little France Crescent, Edinburgh, EH16 4TJ UK; 3 Scottish National Blood Transfusion Service, Edinburgh, UK; 4 School of Medical Sciences, Institute of Medical Sciences, University of Aberdeen, Foresterhill, Aberdeen, UK

**Keywords:** Hypoglycaemia, Impaired awareness of hypoglycaemia, Islet transplantation, Socioeconomic deprivation

## Abstract

**Aims/hypothesis:**

Type 1 diabetes complicated by hypoglycaemia is prevalent in socioeconomically deprived populations. Islet transplantation is of proven efficacy in type 1 diabetes complicated by hypoglycaemia, but it is not known if nationally funded programmes reach the socioeconomically deprived. Our aim was to determine: (1) socioeconomic indices in participants referred to our nationally funded programme; and (2) if metabolic outcomes in our transplant recipients were improved.

**Methods:**

Participants referred (*n* = 106) and receiving transplants (*n* = 18; 32 infusions) were examined with respect to socioeconomic status (deprivation category score) and their ability to work and drive. In participants followed for ≥12 months after transplantation, metabolic and anthropometric measurements (*n* = 14) were recorded pre- and post-transplant (assessed ~1, ~3, ~6 and ~12 months with mixed-meal tolerance tests and 6 day continuous glucose monitoring assessments). Donor data was also examined.

**Results:**

There was a greater prevalence of socioeconomic deprivation in referred and transplant recipients than the general population (*p* < 0.05). Of the transplant recipients, 73% were socioeconomically deprived, 88% did not hold a driver’s license and 94% had reduced ability to work (all *p* < 0.01 vs referred participants). Donors were predominantly obese and included circulatory death donors. At 12 months, 93% of participants who had received transplants had graft function, diminished frequency of hypoglycaemia (10 [4–11] vs 0 [0–2] hypoglycaemic episodes/week), improved awareness of hypoglycaemia (Gold score 7 [5–7] vs 1 [1–2]) and glycaemic control (HbA_1c_: 7.9% [7.2–8.5%]; 63 [55–69] mmol/mol vs 7.2% [6.8–7.5%]; 55 [51–58] mmol/mol), diminished glycaemic lability and decreased central adiposity (all *p* < 0.05).

**Conclusions/interpretation:**

A nationally funded islet transplant programme reaches the socioeconomically deprived and outcomes are significantly improved in this group.

**Electronic supplementary material:**

The online version of this article (doi:10.1007/s00125-015-3554-3) contains peer-reviewed but unedited supplementary material, which is available to authorised users.

## Introduction

Type 1 diabetes is characterised by destruction of pancreatic beta cells by autoimmune processes leading to an absolute requirement for insulin replacement. Hypoglycaemia is the most common side effect of insulin treatment, with severe hypoglycaemia affecting approximately 25% of patients with type 1 diabetes [1, 2], and is associated with increased morbidity and mortality [3, 4]. Repeated episodes of hypoglycaemia are associated with hyperinsulinemia and diminished counterregulatory responses [5, 6], leading to impaired awareness of hypoglycaemia (IAH) [2].

Although there may be an improvement in awareness of severe hypoglycaemia by the avoidance of low blood glucose levels [7, 8], in practice this is difficult to achieve [9]. Allogeneic islet transplantation offers a minimally invasive option for beta cell replacement in people with type 1 diabetes complicated by recurrent severe hypoglycaemia and/or marked glycaemic lability [10], and results in improved awareness of hypoglycaemia [11]. In 2000, the Edmonton protocol for islet transplantation achieved insulin independence in seven consecutive individuals who received high numbers of purified islets under steroid-free immunosuppressive regimens [12].

An integrated UK Islet Transplant Consortium (UKITC) with a commissioned service for Scotland was established between 2008 and 2009 [13]. The consortium was fully funded by the National Health Service and freely available at the point of care. Islet transplantation was endorsed by the National Institute for Health and Care Excellence, with the aim of preventing recurrent severe hypoglycaemia (6), attaining an HbA_1c_ level <7% (<53 mmol/mol), reducing exogenous insulin requirements and quantifying graft function by C-peptide secretion [14]. Achievement of insulin independence was not a primary goal [10]. There is evidence that patients with type 1 diabetes who are socioeconomically deprived have the greatest problems with hypoglycaemia [15], but the degree to which nationally funded services receive referrals or perform transplants in individuals from such groups is not known.

A shared national vascularised pancreas and islet transplant donor allocation scheme exists in the UK. Donor pancreases are equally distributed between solid organ and islet recipients, and pancreases from donors with a high BMI are preferentially offered for use in islet transplantation. The aim of islet transplantation is to achieve a total of ≥10,000 islet equivalents (IEQ)/kg recipient weight within 12 months of the first transplant, including the routine use of islets from donation after cardiac death (DCD).

Our aim was to determine the socioeconomic and demographic indices of patients referred to, and receiving islet transplants in, our nationally funded programme [16] and to evaluate the metabolic outcomes of patients receiving islet transplants at our single UK centre [17].

## Methods

### Organ donors, islet isolation and transplantation procedures

Donor data including sex, ethnicity, age, weight, height, BMI, waist circumference, DCD vs donation after brain death (DBD) status and ischaemic time were recorded. Following digestion with Liberase (Roche, Basel, Switzerland), islets were purified, quality-assessed and product-released at a median time of 36 h from the Scottish National Islet Isolation Laboratory [18] with minimum release criteria [17]. IEQ counts, purity and viability were documented. Islets were transplanted by percutaneous transhepatic delivery into the portal vein under radiological guidance, and recipients received intravenous insulin and heparin infusions perioperatively, followed by low molecular weight heparin for 7 days [17]. All recipients received alemtuzumab 30 mg subcutaneously preceding their first transplant and were maintained on tacrolimus (target level 7–9 μg/l) and mycophenolate mofetil, along with prophylactic trimethoprim/sulfamethoxazole and valganciclovir (in the case of donor or recipient cytomegalovirus positivity) for 6 months.

### Referred participants

Participants were enrolled from November 2009 until May 2014 from our clinic. Detailed personal information (including social and demographic data, employment status, driving status, ethnicity and smoking status) was collected from all referrals. The Deprivation Category (DEPCAT) score divides postcode sectors into seven categories in relation to income, employment, health, housing and education and is a robust measure of socioeconomic status [19]. People with DEPCAT scores of 4–7 have been classified as being socioeconomically deprived [19]. DEPCAT scores for the 5.06 million population in Scotland were derived from the 2001 census [19], and the national prevalence of socioeconomic deprivation was compared with data from referred and transplanted participants. The employment status of the participants was compared with the national figures from the Scottish Household Survey [20]. In the UK, stringent driving regulations exist and individuals with severe hypoglycaemia requiring assistance must declare this to the Driver and Vehicle Licensing Agency (DVLA) and cannot re-apply for their license until 12 months have passed since their penultimate hypoglycaemic event [21, 22]. The proportion of people with a driver’s license in the general population was derived from government statistics [23] and compared with data from our participants.

### Islet transplant recipients

All participants had C-peptide-negative diabetes complicated by recurrent severe hypoglycaemia (≥1 event over the preceding 12 months that required assistance) despite optimised conventional management. All except one participant received transplants for severe IAH; one participant with normal renal function received a transplant for glycaemic variability with progressive complications from diabetic retinopathy. Severe insulin resistance (insulin requirements >0.7 units/kg with daily dose insulin >60 units and HbA_1c_ level <9.0% [75 mmol/mol]) was an absolute contraindication and obesity a relative contraindication for islet transplantation. There were no contraindications to immunosuppressive therapy and renal function was not impaired (GFR >60 ml/min/1.73 m^2^ and albumin excretion rate <300 mg/24 h).

Participants eligible for transplants were listed on the UK Organ Donation and Transplant waiting list after HLA typing and unacceptable HLA antigen definition to avoid immunologically incompatible transplantation. All islet transplant recipients underwent the following assessments at each visit.

#### Assessment visits

Participants were assessed pre-transplant (anthropometric measurements were made on the ward prior to transplant) and then at approximately 1, 3, 6 and 12 months after first transplant as per UKITC protocols. All assessments except the 3 month assessment were made within 1 week before or after the intended time point; at 3 months, the median (interquartile range [IQR]) time was 3.6 (3.3–3.5) months post-transplant by which time eight of the 14 participants had received a second transplant. At the first visit, detailed personal histories were taken and at all visits diabetes control, hypoglycaemia measures, caloric assessments and anthropometry were assessed as outlined below.

#### Diabetes assessments, hypoglycaemia measures and food diaries

Duration of diabetes was calculated at the time of the first islet transplant. Information about insulin therapy, awareness of hypoglycaemia (Gold Score and Clarke Scores ≥4, reflecting IAH) and frequency of severe hypoglycaemia pre-transplant and prospectively post-transplant (over the preceding 1 week period) was collected at all visits. Hypoglycaemia was recorded as a plasma glucose concentration <4 mmol/l [24, 25]. Continuous glucose monitoring systems (CGMS) (iPro2; Medtronic, Northridge, CA, USA ) were used to gain 6 day glycaemic profiles. Over this period, self-reported food diaries were completed.

Complications from diabetes were recorded at baseline, including a history of previous diabetic retinopathy [26], using the Scottish Care Information–Diabetes Collaboration database and hospital records. A history of autonomic neuropathy was diagnosed from investigations and/or from the clinical history, and peripheral neuropathy was measured using a neurothesiometer (Euroenergy, Horwell, UK) [27]. A vibration perception threshold (VPT) score ≥15 indicated peripheral neuropathy [28]. The study was not powered to assess changes in these complications post-transplant.

#### Anthropometric measurements

Participants were weighed to the nearest 0.1 kg (SECA 959 electronic chair scales, SECA, Birmingham, UK). Height, waist (between the lower margin of the last palpable rib and the top of iliac crest [29]), hip circumference (mid-trochanter), left mid-arm and left mid-thigh circumference were recorded to the nearest 0.5 cm. Body fat was estimated by air displacement plethysmography (BOD POD; COSMED USA, Concord, CA, USA).

#### Mixed-meal tolerance tests with blood sampling

Venous blood samples were collected after an 8–10 h overnight fast at 09:00 h. Participants adjusted their insulin therapy as described [30] and a mixed meal of Ensure HP (Abbott, Maidenhead, UK) was administered; sampling was carried out at −15, 0, 30, 60, 90, 120 and 180 min and plasma stored at −80°C. Fasting samples were taken for the measurement of glucose, C-peptide, HbA_1c_, full lipid profile (total and HDL-cholesterol and triacylglycerol), full blood count, liver function tests and antibodies, and stimulated samples for the measurement of glucose and C-peptide. A 90 min C-peptide level >50 pmol/l was evidence of graft function [17].

### Laboratory analyses

Glucose and C-peptide concentrations (Perkin Elmer AutoDELFIA,Wokingham, UK until December 2011 and Siemens Immulite 2000, Erlangen, Germany thereafter) were analysed in a centralised laboratory [17]. HLA testing and all other assays were done at the accredited laboratories at the Royal Infirmary of Edinburgh. Concentrations of haemoglobin, alanine transaminase, alkaline phosphatase, bilirubin, creatinine and urea, total cholesterol, HDL-cholesterol and triacylglycerol were measured in automated analysers (Haemoglobin: XE-5000, Sysmex Co, Kobe, Japan; others: Architect 1600, Abbott Diagnostics, Maidenhead UK) [17]. LDL-cholesterol concentrations [17] and estimated GFR were calculated and white cell and lymphocyte counts were determined using flow cytometry (XE-5000, Sysmex, Kobe, Japan). Inter- and intra-assay coefficients of variability were all <10%.

### Statistical analyses

Beta scores were calculated from HbA_1c_, insulin dose and C-peptide concentrations during the mixed-meal tolerance test (MMTT) [30]. The proportion of time spent in hyperglycaemia, euglycaemia and hypoglycaemia was computed from the CGMS data. Data are reported as median (IQR). Differences between referred and transplanted participants were compared using Fisher’s exact test. A comparison of proportions statistical approach was used to compare national data (socioeconomic status, employment and driving statistics) with the corresponding data in the referred and transplanted participants and accounted for sample size. Multiple linear regression analysis with the independent variables socioeconomic status, participant age, duration of diabetes and presence of insulin pump therapy was performed on the referral population to examine independent associations with islet transplantation.

In the 14 participants who had data up to and including 12 months after first transplant, one-way repeated ANOVA analyses were used with post hoc testing to compare pre-transplant data with outcome data at approximately 1, 3, 6 and 12 months post-transplant. Statistical analyses were performed in Stata 12 (Stata Corporation, College Station, TX, USA). A *p* value <0.05 was considered to indicate statistical significance.

### Ethics approval

Participants provided written informed consent and the study was approved by Lothian Research Ethics Committee, UK and conducted in accordance with the principles endorsed by the Declaration of Helsinki.

## Results

### Referred participants

**Fig. 1:**
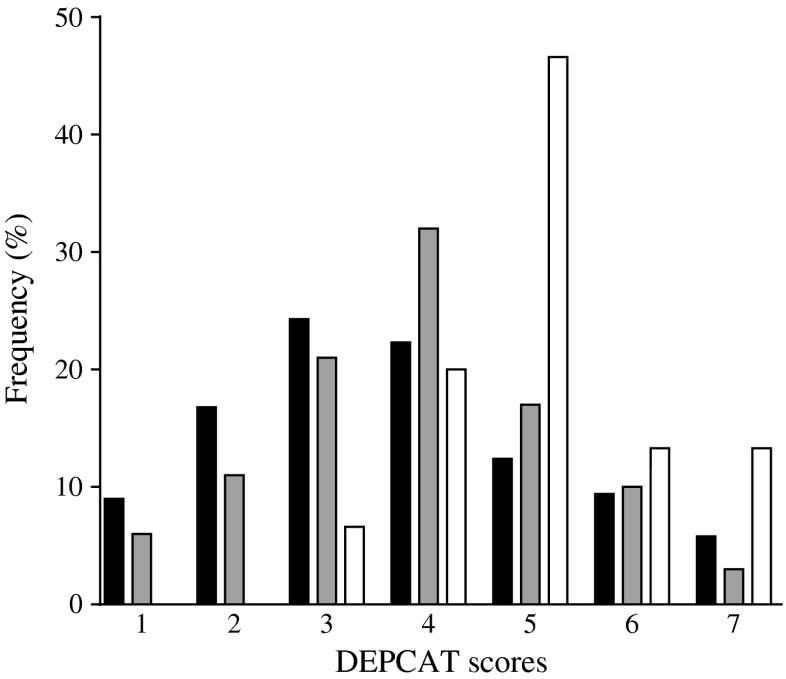
Frequency distribution of DEPCAT scores for participants. Frequency distribution (%) of the general population in Scotland (*n* = 5.06 million; black bars), participants referred for transplant assessment (*n* = 106; grey bars) and participants receiving transplants (*n* = 18; white bars) within each DEPCAT score. *p* < 0.05 for general population vs all groups and referrals vs transplant recipients. Frequency of severe hypoglycaemia is greater in participants with DEPCAT scores ≥4

One hundred and six participants were referred for assessment (57% women) for islet transplantation through our programme in Scotland. The median (IQR) characteristics of the participants were as follows: age 46 (37–53) years, weight 73.3 (63.6–85.0) kg, BMI 25.8 (23.1–29.9) kg/m^2^, insulin dose 0.55 (0.42–0.64) units/kg, HbA_1c_ 8.2% (7.2–9.1%) (66 [55–76] mmol/mol); forty-one per cent were on insulin pump therapy. Sixty-two per cent of referred participants were in lower socioeconomic groups compared with 50% in the general population (*p* = 0.01). The frequency distribution of participants referred for islet transplantation within each DEPCAT score is shown alongside the national data for Scotland in Fig. 1 [19]. Sixty per cent of participants were not employed or had a reduced ability to work, significantly greater than the national figure of 6% registered unable to work due to ill health (*p* < 0.01) [20]. Fifty-eight per cent of participants did not hold a driver’s license, significantly greater than the national figure of 32% (*p* < 0.01) [23].

On multiple linear regression analysis, insulin pump therapy was the only factor associated with being listed for islet transplantation (*p* < 0.05). Referrals for transplantation including exclusions are shown in electronic supplementary material (ESM) Fig. 1.

### Transplanted participants

Eighteen participants all with type 1 diabetes received 32 transplants. Two participants with previous renal transplants were excluded from analyses. Therefore, data on 16 participants (9 women) who received 30 transplants (14 participants with two transplants each; two participants with one transplant each) and who were ≥3 months post-transplant are presented (ESM Fig. 1). Fourteen participants (13 participants with two transplants each; one participant with one transplant) were ≥12 months after the first transplant and sequential outcomes for these participants are also presented. One participant had polyglandular autoimmune disorder and was receiving physiological hormone replacement with hydrocortisone. The median time to second transplant was 3.5 (2.3–4.9) months.

Compared with the general population in Scotland and the referrals, there was a greater prevalence of socioeconomic deprivation in participants who received transplants (73% socially deprived; *p* < 0.05; Fig. 1). In comparison with the referred participants, participants who received transplants demonstrated a decreased ability to work (94%; *p* < 0.01) and drive (88%; *p* < 0.0001). Participants who received transplants were more likely to be on an insulin pump than on a basal-bolus insulin regimen (69%; *p* < 0.05). Baseline data for the transplant recipients are shown (Table 1). Of note, these participants pre-transplant had a high rate of hypoglycaemia with severe IAH as reflected by Gold and Clarke scores (both pre-transplant 7 [5–7]; Table 2) and HbA_1c_ levels at assessment were not notably high (7.9% [7.2–8.5%]; 63 [55–69] mmol/mol; Table 2). Participants were mainly overweight or obese at assessment (five were obese [BMI ≥30 kg/m^2^], six overweight [BMI >25 < 30 kg/m^2^] and five had a normal BMI [BMI >20 < 25 kg/m^2^]) and all participants were of white ethnicity; all had a history of diabetic retinopathy and the majority had peripheral neuropathy and autonomic neuropathy.

**Table 1:** Characteristics of all transplant participants at assessment

Assessment	All participants (*n* = 16)	Participants with data at 1 year (*n* = 14)
Age at first transplant (years)	40 (35–53)	40 (34–52)
Duration of diabetes (years)	31 (27–40)	31 (24–40)
Sex (M:F)	7:9	6:8
White ethnicity (%)	100	100
Smokers %	12.5	14
Socially deprived^a^ (%)	73	64
Inability or reduced ability to work^b^ (%)	94	93
Not driving secondary to diabetes (%)	88	86
Insulin pump therapy (%)	69	71
Episodes of hypoglycaemia per week	9 (4–10)	10 (4–11)
IAH (Gold score ≥4) (%)	94	93
IAH (Clarke score ≥4) (%)	94	93
Autonomic neuropathy (%)	69	64
Diabetic retinopathy (all grades) (%)	100	100
Peripheral neuropathy (%)	75	79

The characteristics of all transplant participants (*n* = 16) and of those with follow-up data for a 1 year period (*n* = 14) are expressed as median (IQR) or percentages, as appropriate

The percentage of participants with IAH were scored by two independent scoring systems (Gold and Clarke scores). Complications from previous diabetes-related retinopathy were ascertained from case notes and retinal eye screening reports, autonomic neuropathy was ascertained from case notes, the clinical history and radiological examinations, and peripheral neuropathy from the case notes including neurophysiological studies and from neuroesthesiometer studies

^a^Patients with DEPCAT scores 4–7 were classed as socially deprived

^b^In all cases, inability or reduced ability to work was secondary to diabetes

**Table 2:** Hypoglycaemia and metabolic assessments of transplant participants over a 1 year period

Metabolic assessment	Pre-transplantation	~1 month	~3 months	~6 months	~12 months	*p* ANOVA
Episodes of hypoglycaemia per week	10 (4–11)	2 (0–3)	1 (0–3)	0 (0–2)	0 (0–2)	<0.0001^a,b,c,d^
Gold score^e^	7 (5–7)	4 (2–6)	3 (2–6)	2 (1–5)	1 (1–2)	<0.0001^b,c,d^
HbA_1c_ (%)	7.9 (7.2–8.5)	7.4 (6.8–7.6)	6.8 (6.2–7.5)	7.0 (6.5–7.2)	7.2 (6.8–7.5)	0.001^b,c^
HbA_1c_ (mmol/mol)	63 (55–69)	57 (51–60)	51 (44–58)	53 (48–55)	55 (51–58)	
Insulin requirements (kg^−1^)	0.52 (0.44–0.57)	0.03 (0.00–0.35)	0.20 (0.08–0.40)	0.31 (0.10–0.49)	0.25 (0.10–0.41)	0.0002^a,b,c,d^
Beta score	0	4 (3–4)	4 (2–5)	4 (3–4)	3 (2–4)	<0.0001^a,b,c,d^
90 min C-peptide (pmol/l)	0 (0–0)	438 (380–550)	697 (294–1,166)	700 (157–878)	667 (303–897)	<0.0001^a,b,c,d^

Measurements were taken pre-transplant and at ~1, ~3, ~6 and ~12 months post-transplant; median (IQR) data for the 14 participants with data up to and including ~12 months post-transplant are shown

One-way ANOVA was performed and recorded with post hoc testing, comparing pre-transplant data with all other time points post-transplant

^a^Denotes significant difference between pre-transplant and ~1 month values

^b^Difference between pre-transplant and ~3 month values

^c^Difference between pre-transplant and ~6 month values

^d^Difference between pre-transplant and ~12 month values

^e^The Gold score was taken in participants who had experienced ≥1 episode of hypoglycaemia since their last assessment

### Donor data

All except one donor pancreas was processed by the Scottish Islet Isolation Laboratory. Detailed donor data are shown in ESM Table 1. IEQs transplanted were 9,355 (7,695–10,741) IEQ/kg, with high purity 84 (76–89)% and viability 94 (91–96)%. Donor pancreases used were predominantly DBD (83%) with longer cold ischaemia times (9.5 [8.5–10.0] h) than those of DCD donors (8.0 [6.5–8.2] h; *p* < 0.01). Thirteen of the donors were obese, 11 overweight and six had normal BMI.

### Transplant outcomes

#### Metabolic control, awareness of hypoglycaemia, diabetes-related complications and lifestyle measures

**Fig. 2:**
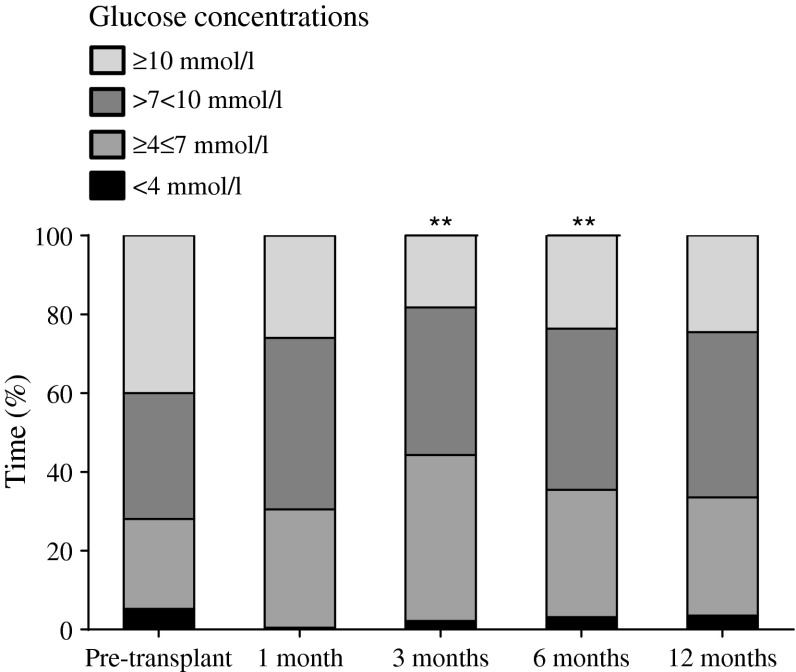
CGMS recordings pre-transplant and at ~1, ~3, ~6 and ~12 months post-transplant. CGMS recordings were carried out for 6 days prior to MMTT assessments. Proportion of time spent in hypoglycaemia (plasma glucose level <4 mmol/l) or hyperglycaemia (plasma glucose level ≥10 mmol/l) was reduced by ~3 and ~6 months post-transplant vs pre-transplant, as assessed by one-way repeated ANOVA analyses with post hoc testing (**p < 0.01)

Islet transplantation resulted in significant graft function post-transplantation as reflected by the stimulated C-peptide concentrations that were maintained over the 1 year period (Table 2). Transplantation resulted in significantly reduced episodes of hypoglycaemia and improved awareness of hypoglycaemia, with a reduction in insulin requirements and an improved beta score of ≥3 at all assessment time points post-transplantation over the 1 year period (Table 2). Improved glycaemic control as reflected by the HbA_1c_ level was apparent at ~3 and ~6 months after the first transplant and was concordant with the CGMS profiles, which showed significantly diminished hypoglycaemia and hyperglycaemia at ~3 and ~6 months post-transplant (*p* < 0.01; Fig. 2). The participant who developed graft failure (ESM Table 1) demonstrated improved metabolic control at 12 months post-transplant vs pre-transplant: time spent in hypoglycaemia (measured by CGMS) was <2% vs 7%; HbA_1c_ level was 7% (53 mmol/mol) vs 7.4% (57 mmol/mol); and the frequency of severe hypoglycaemia was diminished with no recorded episodes 12 months post-transplant vs four episodes per week pre-transplant. Ten of the 14 participants had achieved insulin independence (with plasma glucose levels <10 mmol/l pre- and post-meal) for a range of 1–12 months by 1 year. There was no progression of diabetic retinopathy and one individual demonstrated improvement in their background diabetic retinopathy, and there was no evidence of progression of autonomic neuropathy. There was a nonsignificant decrease in VPT at 12 months post-transplant: 15 (5–16) vs 11 (4–15) volts (*p* = 0.10).

Of the 12 participants who had previously failed to renew their driver’s license secondary to hypoglycaemia, four went on to regain their license following assessments by the DVLA. Five of the 14 participants who were working part time were able to increase their work commitments by 100 (63–200)% post-transplantation, resulting in an average increase in income of 80%.

#### Immunological, haematological and biochemical indices

Of the 30 transplants analysed, matching for HLA-A, -B and -DR was as follows: 1 mismatch (MM), *n* = 1; 2 MM, *n* = 2; 3 MM, *n* = 4; 4 MM, *n* = 13; 5 MM, *n* = 6; and 6 MM, *n* = 4. In first transplant cases, 11 of 16 were negative for HLA class I and class II IgG antibodies (LABScreen, One Lambda (One Lambda, Canoga Park, CA, USA)), four were positive for nondonor-specific class I and/or class II antibodies and in one case weak donor-specific class I antibodies were present. For second transplant cases, eight of 14 were negative for HLA class I and class II IgG antibodies (LABScreen, One Lambda) and six were positive for nondonor-specific class I and/or class II antibodies.

Three participants had increases in donor-specific HLA antibodies after their first transplant, indicative of an active immune response against the graft. In two cases these HLA antibodies were present at a low level pre-transplant and following the initial rise they returned to pre-transplant levels by 3 months post-transplant. In all three cases, the rise in HLA donor-specific antibodies did not coincide with any loss of graft function. In one of the three participants there was graft failure as outlined in the complications section.

Post-transplant there was a fall in haemoglobin levels at each assessment time point including at 12 months (*p* < 0.001); there was an initial fall in white cell count and lymphocyte count (*p* < 0.001) but these had both recovered by 12 months; no change in platelet count was observed. There was a trend for estimated GFR to decrease post-transplant (*p* = 0.06), and serum creatinine increased significantly and remained elevated at each assessment (baseline to 12 months: 72 (64–83) to 93 (81–106) μmol/l, respectively; *p* < 0.001), although this was of no clinical consequence. No change in lipid concentrations (total cholesterol, HDL-cholesterol and calculated LDL-cholesterol and triacylglycerol) and liver function tests (alkaline phosphatase, alanine transaminase and bilirubin concentrations) were recorded during follow-up.

#### Anthropometric changes

Pre-transplant, nine of the 14 participants were obese or overweight with a median BMI of 26.7 kg/m^2^ (Table 3). A reduction in weight, percentage fat mass and arm circumference was seen by 6 months and was still evident at 12 months post-transplant. Waist and hip circumference decreased significantly by 12 months post-transplant. Energy intake to prevent severe hypoglycaemia was reduced at each assessment period post-transplant, although total energy intake was not significantly reduced (Table 3).

**Table 3:** Anthropometric and food intake measurements for hypoglycaemia of transplant recipients over a 1 year period

Anthropometric and caloric assessment	Pre-transplantation	~1 month	~3 months	~6 months	~12 months	*p* ANOVA
Weight (kg)	74.0 (64.4–81.7)	73.9 (62.9–80.3)	74.5 (61.5–78.9)	72.9 (57.5–79.0)	70.1 (59.0–78.3)	<0.0001^c,d^
BMI (kg/m^2^)	26.7 (23.5–29.8)	26.4 (23.6–29.3)	26.4 (23.7–28.7)	25.7 (21.1–28.2)	25.2 (21.3–26.6)	<0.0001^c,d^
Fat mass (%)	31.4 (20.3–35.9)	29.3 (21.1–35.2)	27.8 (20.9–34.6)	25.8 (16.8–34.5)	23.5 (16.3–32.3)	<0.0001^c,d^
Waist circumference (cm)	85.5 (78.5–95.0)	85.0 (79.5–95.2)	82.0 (79.5–94.0)	81.5 (75.0–92.0)	79.0 (76.0–94.0)	0.0004^d^
Hip circumference (cm)	95.0 (90.5–101.5)	97.0 (91.0–104.5)	94.5 (88.0–99.5)	94.0 (88.5–101.5)	90.0 (86.5–100.5)	0.01^d^
Left mid-arm circumference (cm)	30.0 (28.0–32.0)	30.0 (28.0–32.0)	30.0 (28.0–30.5)	29.5 (27.0–30.5)	29.0 (27.0–30.0)	0.001^c,d^
Left thigh circumference (cm)	53.0 (46.0–59.0)	52.0 (46.0–55.0)	52.0 (46.0–58.0)	51.0 (47.0–57.0)	50.0 (46.0–55.0)	0.37
kJ for hypoglycaemia (week^−1^)	4,665 (3,105–8,954)	456 (113–1,402)	573 (146–1,623)	0 (0–423)	0 (0–293)	<0.0001^a,b,c,d^
Total kJ (day^−1^)	7,858 (7,510–9,318)	7,531 (7,355–8,029)	7,184 (6,184–8,134)	7,251 (6,205–7,820)	6,971 (5,912–7,707)	0.08

Anthropometric and dietetic measurements were taken pre-transplant and at ~1, ~3, ~6 and ~12 months post-transplant; median (IQR) data for the 14 participants with data up to and including ~12 months post-transplant are shown

One-way ANOVA was performed with post hoc testing, comparing pre-transplant data with all other time points post-transplant

^a^Denotes significant difference between pre-transplant and ~1 month values

^b^Difference between pre-transplant and ~3 month values

^c^Difference between pre-transplant and ~6 month values

^d^Difference between pre-transplant and ~12 month values

#### Complications

One participant who received two islet transplants separated by 2 months had complete graft failure (C-peptide level <50 pmol/l) 4 months after their first transplant. In this patient, de novo transient HLA antibodies directed at an HLA mismatch from the first islet graft were detected previously but they were not coincident with graft failure. One participant developed tinnitus on tacrolimus, and sirolimus was substituted. One participant developed gastritis, and one developed neutropenia secondary to the immunosuppression and required a single course of growth colony stimulating factor. There were no surgical complications and no cases of carcinoma in the short time of follow-up in this programme.

## Discussion

The results from our single, health-service funded centre in Scotland confirms that islet transplantation significantly reduces severe hypoglycaemia [25] and exogenous insulin requirements, and improves diabetes control (as reflected by the HbA_1c_ and CGMS profiles, with evidence of C-peptide secretion) [17]. These findings are consistent with those from other centres [10, 31–33] and previously published UK outcomes [17]. Importantly for a nationally funded service, the results show that the majority of participants both referred and receiving transplants were from lower socioeconomic groups than the general population [19], with a diminished ability to work and drive. Furthermore, patients receiving transplants were the most severely affected of the referrals in terms of socioeconomic, employment and driving status.

The prevalence of type 1 diabetes is not associated with social class [34, 35], showing equal representation in all socioeconomic groups. However, severe hypoglycaemia complicating type 1 diabetes occurs most frequently in those of lower socioeconomic status [15]. This study is the first to show results related to socioeconomic status and suggests that a programme funding by a health service drives referrals and islet transplantations in patients with type 1 diabetes from such groups. Living in Scotland is associated with socioeconomic deprivation [16, 36], and there may be a greater degree of socioeconomic deprivation in our participants than transplant recipients in other centres in the UK [17], although further analyses are required to confirm this. The age of recipients and duration of diabetes was in line with that in other studies [10, 37]. Although some of the participants who received transplants were on basal-bolus insulin regimens, insulin pump therapy was associated with being listed for islet transplantation, perhaps reflecting a staged approach to diabetes management in many patients.

Although not powered to examine quality of life measures, employment, personal income and the ability to drive appeared to improve following transplantation and >70% of participants achieved insulin independence, consistent with the findings of other studies [10]. Studies that have more formally examined quality of life indices have found improvements [38] and we await further prospective studies in this field.

Our transplant recipients were mainly overweight pre-transplant and the weight loss post-transplant was consistent with that in other observational studies with <18 months follow-up [32, 37]. Unlike many studies, none of our participants were treated with glucagon-like peptide-1 receptor agonists, which may confound the results as they have central inhibitory effects on appetite [37]. We have extended previously published observations to show a decrease in central and upper body fat as measured by gold standard techniques post-transplant [37]. The aetiology of the post-transplant weight loss is likely to be multifactorial, including increased energy expenditure related to both increased activity and postprandial thermogenesis secondary to endogenous insulin secretion, as well as a central effect of immunosuppressive therapy on appetite. It is possible that participants may have underestimated their energy intake using the food diaries and more accurate assessments of energy intake would be useful [39]. Our study is limited by the fact that the participants were on differing quantities of exogenous insulin and it would be more controlled to study transplant recipients that are insulin independent to better understand the changes in energy balance post-transplantation.

Notably, no major complications have been demonstrated and the changes in haematological indices and renal function secondary to the immunosuppression have been previously reported [32]. In contrast to other studies, we did not see a rise in triacylglycerol concentrations post-transplant, perhaps because immunosuppression regimens differed.

The occurrence of donor-specific HLA antibodies in three participants was transient in each case and not related to coincident graft dysfunction or C-peptide response, although we cannot comment on the impact that this has on long-term graft survival [40].

The predominance of higher BMI donor pancreases isolated for islet transplantation reflects that the aims of the shared vascularised pancreas and islet transplantation service have been met. The second islet transplant was also given ~3 months from the first transplant in line with the goals of the programme. Despite not achieving a target islet mass (IEQ ≥10,000/kg) in many of our patients, our short-term clinical outcomes are comparable to those of other centres and reflect the pragmatic approach that it has been necessary to adopt in the face of an ongoing shortage of organ donors and an overweight recipient population. DCD donors, in common with other programmes [10], were utilised to good effect. Although no participant received islet transplants from two DCD donors (precluding an analysis of DCD status alone), participants receiving two islet transplants including one from a DCD donor had equivalent stimulated C-peptide concentrations to participants receiving islet transplants from DBD donors alone. This finding is consistent with the observation that islets from DCD donors can be used effectively. The relatively good C-peptide responses that have been attained may be due to a relatively young donor age, although other factors may be important. The advantage of assessing measures from a single transplant and isolation centre is that participant assessments and the isolation procedures are less prone to variability. Our outcomes, with stimulated C-peptide readings reflecting a functioning graft and stable glycaemic control as reflected by the CGMS recordings with beta scores ≥3, are consistent with the aims of the programme for predominantly abrogating hypoglycaemia [41]. The deterioration in glycaemic control >6 months post-transplant is in line with the outcomes from other programmes [32].

In conclusion, the islet transplantation outcomes from our single, nationally funded health centre in Scotland demonstrate that referrals and transplantations are predominantly from socioeconomically deprived groups with low employment and a decreased ability to drive secondary to their hypoglycaemia. Islet transplantation reduced the frequency of hypoglycaemia and improved awareness of hypoglycaemia and glycaemic control. Transplantation also diminished insulin requirements and decreased central obesity and fat mass. Furthermore, there was evidence of endogenous insulin secretion post-transplantation. Our studies indicate that quality of life may be improved post-transplantation, as assessed by driving status, an increased ability to work and increased personal income; however, further prospective studies are required to demonstrate long-term socioeconomic benefit.

## Electronic supplementary material

Below is the link to the electronic supplementary material.

ESM Fig. 1 (PDF 11.8 kb)

ESM Table 1 (PDF 24.7 kb)

## Abbreviations

CGMS: Continuous glucose monitoring systems
DBD: Donation after brain death
DCD: Donation after cardiac death
DEPCAT: Deprivation category
DVLA: Driver and Vehicle Licensing Agency
IAH: Impaired awareness of hypoglycaemia
IEQ: Islet equivalents
IQR: Interquartile range
MM: Mismatch
MMTT: Mixed-meal tolerance test
UKITC: UK Islet Transplant Consortium
VPT: Vibration perception threshold
