# Supplementary material for: Islet transplantation from a nationally funded UK centre reaches socially deprived groups and improves metabolic outcomes
Source: Diabetologia. 2015 Mar 26;58(6):1300–8. doi: 10.1007/s00125-015-3554-3 (PMC4415991; doi:10.1007/s00125-015-3554-3)
Supplement: Supplementary file 1 — (PDF 11.8 kb) [file 125_2015_3554_MOESM1_ESM.pdf]

ESM Fig. 1 Participants assessed for islet transplantation

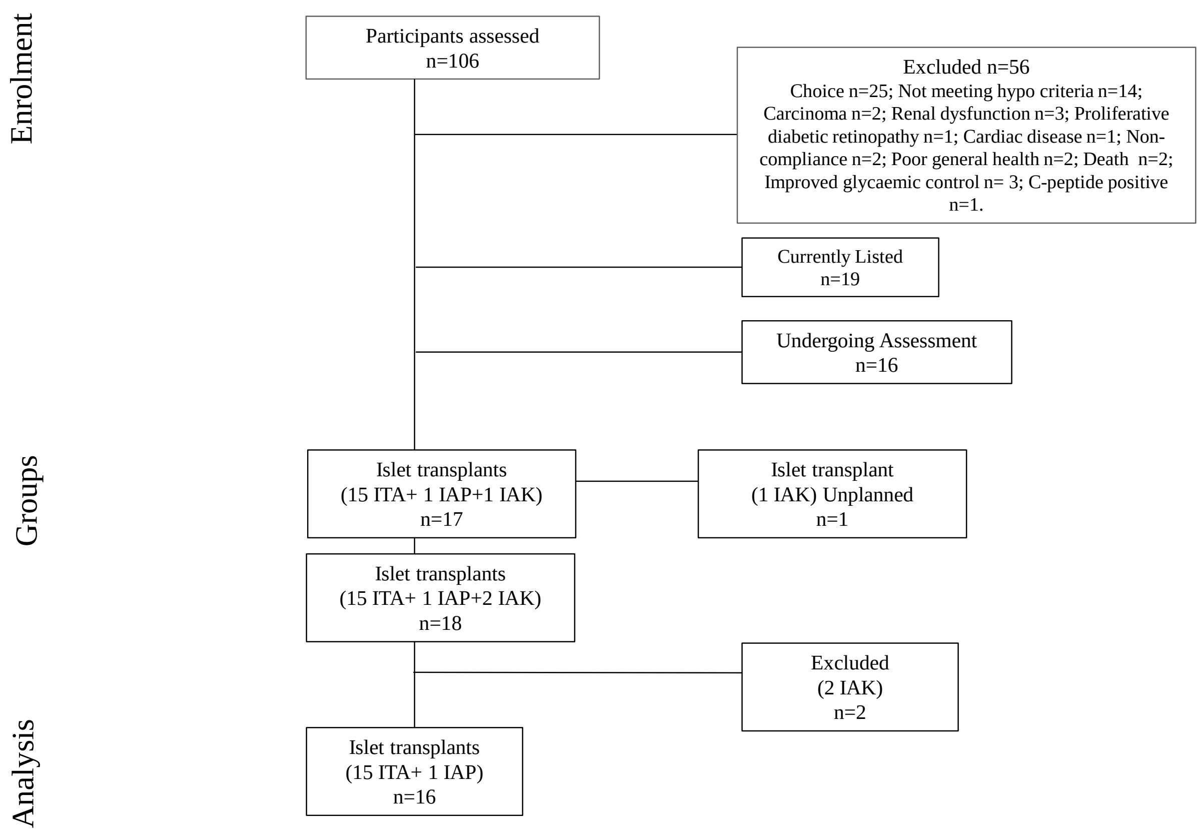

Referrals for transplantation: those excluded, listed, undergoing assessment and in receipt of islet transplants are shown. Two participants listed had received one islet transplant each. IAK participants were excluded from analysis as shown. The majority of participants excluded continue to have problematic hypoglycaemia.

IAK – islet after kidney; IAP – islet after pancreas; ITA – islet transplant alone.
