# Supplementary material for: Islet transplantation from a nationally funded UK centre reaches socially deprived groups and improves metabolic outcomes
Source: Diabetologia. 2015 Mar 26;58(6):1300–8. doi: 10.1007/s00125-015-3554-3 (PMC4415991; doi:10.1007/s00125-015-3554-3)
Supplement: Supplementary file 2 — (PDF 24.7 kb) [file 125_2015_3554_MOESM2_ESM.pdf]

ESM Table 1 Donor data including islet quality and numbers isolated

Participant	Total IEQ/kg*	Purity (%)	Viability (%)	DCD/DBD[1,2]	Ischaemic time (hours)	M/F[1,2]	Age (years)	Weight (kg)	BMI (kg/m^2)
**1**	9,369	73	95.5	DBD, DCD	8.0	F,F	32	66.5	28
**2**	9,341	90	95.5	DCD, DBD	7.5	M,M	39	72.7	24
**3**	7,850	75	96	DBD, DBD	8.0	M,M	56	98.5	30
**4**	11,539	85	93.5	DBD, DBD	8.5	F,F	52	88	37
**5**	9,424	92.5	90	DBD, DBD	10.8	F,M	39	77.5	28
**6**	8,573	75	96.5	DBD, DBD	10.3	M,F	46.5	85	29
**7**	16,261	77	94.5	DBD, DBD	9.8	M,F	53	80	31
**8**	9,158	80	96	DBD	10.0	M	17	102	33
**9**	7,229	81	89.5	DBD, DCD	8.5	F,F	32	85	35
**10**	11,584	92.5	92	DCD, DBD	8.2	M,F	51	85	28
**11**	13,254	95	95.5	DBD, DBD	8.5	M, F	47	79	24.5
**12**	9,875	86.25	85.5	DBD, DBD	10.2	M,F	42.5	102.5	34
**13**	7,059	84.5	93.5	DCD, DBD	8.5	F,M	33.5	89.1	26
**14**	6,847	74.25	89	DBD, DBD	8.8	M,F	36	105	37
**15**	6442	85	93	DBD	8.0	M	58	108	34
**16**	10,475	83	96	DBD,DBD	9.0	M,M	41	90	28
Median (IQR)	*9355 (7,695 -10,741)*	*84 (76-89)*	*94.0 (91-96)*	*25 DBD, 5 DCD*	*8.6 (8.1-10.0)*	*14F, 16M**	*45 (36-52.0)*	*86.5 (79.3-101.1)*	*29.5 (27.6-34.0)*

*Total IEQ received were expressed per kg body weight of the recipient; where two transplants were received, donor data (purity, viability, ischaemic time, age, weight and BMI) was averaged. DBD vs. DCD status and gender of donor are as indicated for [1]first and [2] second transplants. DCD status was associated with statistically lower ischaemic times compared to DBD status: 8.0(6.5-8.2) vs. 9.5(8.5-10.0) hours; p=0.003. Twenty-nine donors were white and 1 of black ethnicity; Participant 13, developed graft failure. Median (IQR) data are shown.
